# Use of Period Analysis to Timely Assess Five-Year Relative Survival for Patients with Ovarian Cancer from Taizhou, Eastern China

**DOI:** 10.3390/jcm12103480

**Published:** 2023-05-16

**Authors:** Xin Bing, Huijun Lei, Xiaojiao Zhao, Yongran Cheng, Liangyou Wang, Jun Yang, Mingzhi Xu, Chenhuan Yu, Tianhui Chen

**Affiliations:** 1 Department of Cancer Prevention, Zhejiang Cancer Hospital, Hangzhou 310022, China; 2021112012152@stu.hznu.edu.cn (X.B.); yc17662@umac.mo (H.L.); 2021112012133@stu.hznu.edu.cn (X.Z.); 2 School of Public Health, Hangzhou Normal University, Hangzhou 311121, China; gastate@zju.edu.cn; 3 Institute of Basic Medicine and Cancer (IBMC), Chinese Academy of Sciences, Hangzhou 310022, China; xumingzhi@zju.edu.cn; 4 School of Public Health, Hangzhou Medical College, Hangzhou 310013, China; chengyr@zjams.com.cn; 5 Department of Non-Communicable Chronic Disease Control and Prevention, Taizhou Center for Disease Control and Prevention, Taizhou 318000, China; 13566877151@163.com; 6 Department of General Medicine, Zhejiang Cancer Hospital, Hangzhou 310022, China; 7 Department of Preventive Medicine, School of Medicine, Ningbo University, Ningbo 315211, China

**Keywords:** ovarian cancer, five-year relative survival, cancer registry, period analysis, eastern China

## Abstract

Objective: Ovarian cancer is a deadly gynecologic malignancy with a poor prognosis. It is essential to evaluate the early detection and screening programs of ovarian cancer via timely assessment of long-time survival, particularly in China where those data are incredibly limited. Here, we aimed to provide timely and accurately assessment of long-term survival estimate of ovarian cancer patients from eastern China. Methods: Data of 770 ovarian cancer patients diagnosed between 2004–2018 were obtained from four cancer registries in Taizhou, eastern China, were included. We used period analysis to calculate five-year relative survival (RS) of aforementioned ovarian cancer patients for overall and the stratification by age at diagnosis and region. Results: Our findings demonstrated that the overall five-year RS for ovarian cancer patients in Taizhou between 2014 and 2018 was 69.2%, while urban areas were higher compared to rural areas (77.6% vs. 64.9%). We also observed a significant age gradient with the five-year RS decreasing from 79.6% for age group < 55 years to 66.9% for age group > 74 years. Furthermore, we identified a clear upward trend of five-year RS over the study period, both overall and stratified by region and age at diagnosis. Conclusion: This is the first study in China using period analysis to provide the most up-to-date five-year RS for ovarian cancer patients from Taizhou, eastern China, which reaches 69.2% during 2014–2018. Our results provide valuable information for timely assessment of early detection and screening programs for ovarian cancer in eastern China.

## 1. Introduction

Ovarian cancer ranks as the seventh most prevalent cancer amongst women, accounting for approximately 4% of female-associated cancers worldwide [1]. The lifetime risk of developing ovarian cancer in women is 1 in 75, and the probability of dying from the disease is 1 in 95 [2]. Incidence remains different across age and ethnic groups, with rates being higher in less developed countries (approximately 70%) [3] and more stable in Western countries [4]. According to the latest GLOBOCAN 2020 data, 313,959 new cases of ovarian cancer and 207,252 deaths will occur worldwide in 2020, with an age-standardized incidence rate of 6.6/10^5^ and a mortality rate of 4.2/10^5^ [5]. China’s morbidity (4.54/10^5^) and mortality (2.77/10^5^) are low, and they are currently lower than the United States, Russia, Japan, India and the global average [6]. Due to the lack of effective early detection strategies and unfavorable anatomical situations, ovarian cancer is usually detected at an advanced stage with a poor prognosis. Therefore, ovarian cancer has the lowest survival among all gynecologic cancer sites [4]. The global five-year overall relative survival is generally between 30–40%, with only a minimal increase (2–4%) since 1995 [7,8]. 

Relative survival (RS) is an important indicator of long-term survival that can reflect cancer prevention effectiveness and prognosis in a specific region. Long-term survivals, such as the five-year and ten-year survivals, which are important indicators to evaluate the prognosis of cancer and reflect the extent of the disease’s harmfulness, have been widely used to monitor the progress of cancer diagnosis and treatment [9,10]. The calculation methods of five-year RS of cancer patients include cohort, complete, and period analysis approach. Traditional cohort approach generally requires the use of five years of follow-up data, which means that a minimum of five years of survival estimates (even an extended period for data compilation, computation, and disclosure) will be delayed, and survival estimates will be significantly lower than real survival. In fact, period method is fundamentally different from traditional cohort method. Period analysis method estimate survival without requiring five years of follow-up data, making it superior to the traditional cohort method in terms of timeliness and accuracy of survival analysis [11,12]. Meanwhile, the period analysis method is widely implemented in Western populations as the “gold standard” for long-term survival assessment for patients with malignant cancers [13,14].

Our group has systematically demonstrated for the first time in China that the period analysis method surpasses the traditional cohort method. It provides newer and more accurate estimates of long-term survival, stratified by sex, region, age at diagnosis, and cancer site [15]. During 2009–2013, our group actually observed a five-year true survival of 56.3% for ovarian cancer patients in Taizhou, eastern China, while the five-year RS estimated using cohort method and period analysis method were 51.9% and 55.4%, respectively. The results of period analysis method can be shown to be closer to the true survival [15]. However, limited implementation of period analysis was reported in China, especially for ovarian cancer assessment.

The objective of this study is to furnish up-to-date estimates of the overall and stratified five-year RS of ovarian cancer patients in the Chinese population (2014–2018) by using data from Taizhou City, eastern China, for period analysis, taking advantage of the population-based cancer registry. We also assessed the trends of five-year RS during 2004–2018.

## 2. Materials and Methods 

### 2.1. Data Sources

Taizhou is located in East China and on the central coast of Zhejiang Province, with a population of 6.601 million. Zhejiang Health Surveillance zone was established in 2001 and distributed in 30 counties (cities, districts) of 11 cities in Zhejiang Province [16]. The data of ovarian cancer came from the Taizhou cancer registry. Based on the inclusion criteria that the proportion of cases with only ‘death certificate only’ was less than 13%, and data from four of the nine counties (Luqiao District, Yuhuan District, Xianju County and Wenling City) were used for subsequent analysis [15].

The ovarian cancer patients diagnosed from 1 January 2004 to 31 December 2018 and followed up to 31 December 2018 were selected as the study subjects. We included 912 cases in the database according to the ovarian cancer ICD-10 code C56. Fifty-six cases of lost follow-up, 24 cases of unknown records, and 13 cases of missing follow-up time were deleted. Then, we further reviewed the data and deleted 49 cases of logic errors. Finally, 770 eligible patients from 2004 to 2018 were included for further analysis.

### 2.2. Statistical Analysis

Differences in the distribution of patients’ basic characteristics were statistically analyzed between 2004–2008, 2009–2013 and 2014–2018 using the categorical variables *χ^2^* test, and *p* < 0.05 was considered statistically significant. We used the period analysis method to calculate the five-year RS of ovarian cancer patients during 2014–2018. The study cohort comprised two groups: those who received a new diagnosed in 2014–2018 (in the period of interest) and those who were diagnosed in 2009–2013 (before the period of interest) and survived in 2014–2018 (in the period of interest). The follow-up period was 2014–2018. The method needs to process the left censored data diagnosed in 2009–2013 and the right censored data that are still alive after 2018. This approach involves organizing the data to construct a life table and further calculating the conditional one-year RS *(S_i_*) of the i year of follow-up, which is expressed as:

$$S_{i}=1-\frac{d_{i}}{n_{i}-c_{i}/2}$$

In the formula, $n_{i}$ represents the population at the beginning of the i year, $d_{i}$ represents the number of deaths at the end of the i year, and $c_{i}$ represents the number of deletions in the i year. The real survival $\bar{S_{k}}$ of k years is multiplied by the conditional one-year RS of k years, which is expressed as:

$$\bar{S_{k}}=\prod_{i=1}^{k}S_{i}$$

RS is the ratio of the real survival to expected survival, expressed as:

$$R_{i}=\frac{\bar{S_{k}}}{S_{k}^{*}}$$

When calculating the five-year RS, k = 5. Where $\bar{S_{k}}$ represents the real survival, and $S_{k}^{*}$ represents the expected survival. The expected survival was calculated by the Ederer II method. The point estimation of RS and its standard error (SE) were calculated by the Greenwood method. The above calculation is realized using the “PeriodR” package in R version 3.1.3 software (R Foundation for Statistical Computing, Vienna, Austria) [17].

## 3. Results

### 3.1. Basic Information for Patients with Ovarian Cancer

Table 1 shows the basic characteristics of ovarian cancer patients. It consisted of 770 individuals diagnosed with ovarian cancer, and the number of patients increased from 2004 to 2018. With more patients in rural areas than in urban areas registered, there are 585 (76%) and 185 (24%) patients in each group (*p* < 0.0001). Although an increase in the number of registered ovarian cancer cases was observed in both regions, the rise was more pronounced in rural areas. The total average age of diagnosis was 54.4 years. From 2004–2008 to 2014–2018, the age of diagnosis became younger. The highest proportion of patients was aged > 74 years (30.6%), and the lowest proportion of patients was aged 55–64 years (21.3%). The number of patients in each age group increased yearly over time in all three periods. The age distribution difference was not significant during 2004–2018 (*p* = 0.5621).

### 3.2. Five-Year RS of Ovarian Cancer Patients during 2014–2018

Using period analysis, we assessed the five-year survival of ovarian cancer patients from 2014 to 2018 in Taizhou, eastern China. During 2014–2018, we found an overall five-year RS of 69.2% for ovarian patients (Table 2). Patients residing in urban areas exhibited a higher five-year RS than those in rural areas (77.5% vs. 64.9%). Additionally, we noted a significant age gradient in the five-year RS. From 79.6% at the age of diagnosis < 55 years to 66.9% at the age > 74 years, the five-year RS decreased with a growing age. 

### 3.3. Five-Year RS Trends during 2004–2018

**Figure 1:**
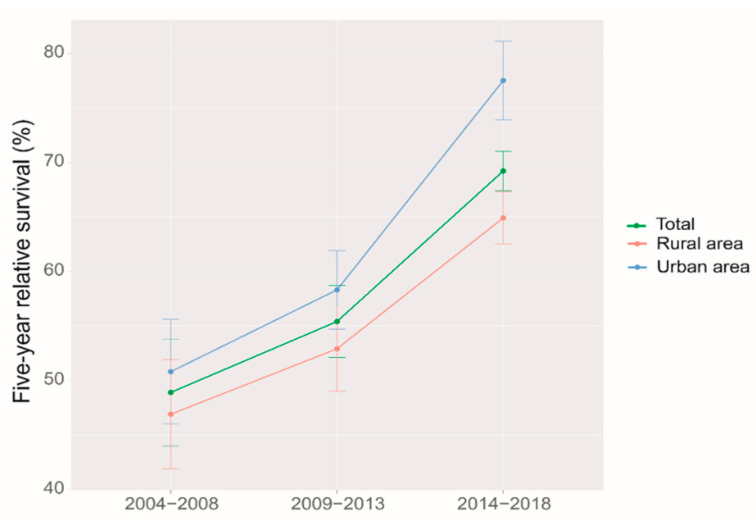
Five-year relative survival for patients with ovarian cancer by region in 2004–2008, 2009–2013 and 2014–2018.

**Figure 2:**
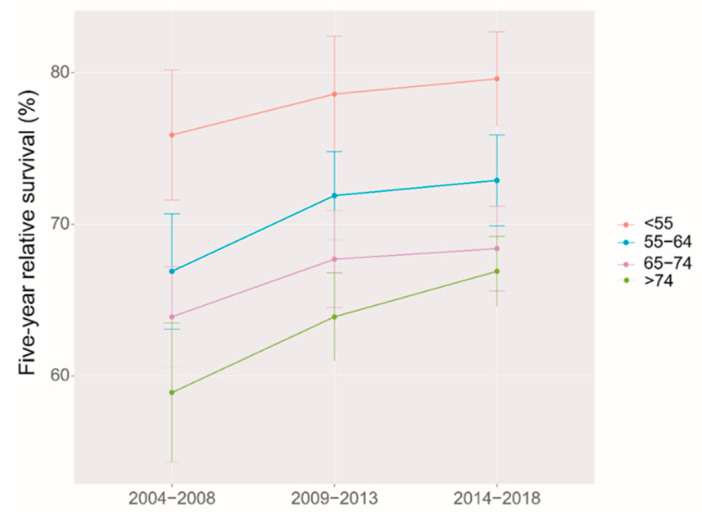
Five-year relative survival for patients with ovarian cancer by age at diagnosis in 2004–2008, 2009–2013 and 2014–2018.

We identified a clear upward trend of five-year RS over the study period, both overall and stratified by region and age at diagnosis during 2004–2018. (Figure 1 and Figure 2). We also found that the trend of increasing five-year RS stratified by age at diagnosis slowed down after the 2009–2013 period, especially in the 65–74 age group, which largely leveled off. The five-year RS gap between the age 65–74 years, and >74 years groups were narrowed down during 2014–2018 compared to those during 2009–2013.

## 4. Discussion

It was the first time that period analysis implemented in China to evaluate the overall latest five-year RS of ovarian cancer patients in Taizhou, Eastern China, which reached 69.2% during 2014–2018. The five-year RS in urban areas was higher than that in rural areas (77.6% vs. 64.9%). Furthermore, a significant age gradient was observed for the five-year RS, which decreased from 79.6% at the age of diagnosis < 55 years to 66.9% at the age > 74 years. During 2004–2018, the overall five-year RS and the RS stratified by region and age at diagnosis showed an upward trend. 

Our study found that the five-year RS for Taizhou reached 69.2% in 2014–2018, higher than 55.2% reported for Zhejiang Province in 2005–2010 [16] and 39.1% reported for China in 2012–2015 [18]. However, our results are plausible for the following three reasons. First, the data reported for Zhejiang Province (2005–2010) is eight years ahead of our data (2014–2018) at 55.2%. It is well known that, as the level of treatment has improved in recent years, the survival rate of ovarian cancer patients is probably significantly higher [19,20,21], and access to early screening programs and medical insurance for patients with ovarian cancer is also increased [22,23]. Secondly, the report of 55.2% in Zhejiang Province from 2005–2010 was computed using the cohort approach, which tends to underestimate five-year RS when compared to the rates computed using the period approach, as evidenced by our team’s analysis of five-year RS between 2009–2013 [15]. Thirdly, the reported five-year RS of 39.1% for 2012–2015 in China is actually a prediction, rather than an estimation due to the limited data available for the study (only 17 cancer registries in China provided information on cancer patients diagnosed prior to the end of 2013 and followed up until 2015 in this study [18], so survival data after 2013 can only be predicted) and may be significantly underestimated. Overall, our study utilized data from eastern China, where there is an advanced healthcare system and qualified data, leading to typically much higher rates when compared to data from 17 cancer registries with different health care systems [18].

The five-year RS was higher in urban areas than in rural areas (77.6% vs. 64.9%), which may be related to the factors, such as economics, health awareness of the population, and allocation of medical resources [16]. Wu et al. demonstrated significant differences in RS between different economic levels [24]. The overall awareness rate of Zhejiang residents of core cancer prevention and treatment is 78.4%, with urban and rural areas being one of the factors affecting the awareness rate, and urban residents having a higher knowledge of cancer than rural areas [25]. Educating women about ovarian cancer and making ovarian cancer diagnosis more accessible to them could help improve ovarian cancer survival rates [26,27]. Quality of care and expertise, as well as treatment by gynecologic oncologists, have also been shown to impact survival [28,29]. Efforts are still needed to better understand the reasons for the urban–rural discrepancy and therefore to eliminate the disparity.

The age and survival of ovarian cancer patients are closely related, as in most cancers [30]. The five-year RS in our study showed a transparent age gradient with a significant decrease in survival with increasing age at diagnosis, consistent with the results from 2005–2010 in Zhejiang Province [16]. Japan, the United States, and Europe showed a similar trend in five-year RS [31]. Older women are more likely to have advanced ovarian cancer at the time of initial diagnosis and have a greater proportion of poorly diagnosed histological subtypes than younger women [32,33] In addition, younger patients often have more aggressive intentions toward treatment than elderly patients. Compared with young ovarian cancer patients receiving more combination treatment, elderly patients are more inclined to monotherapy, which also affects their survival [34]. 

We also found that the five-year overall RS and RS stratified by region and age trended upwards and increased rapidly during 2004–2018. This is reasonable. Firstly, the Chinese government has invested heavily in the healthcare system over the decades, and deepening healthcare reform is a major reason [35]. Secondly, advances in clinical treatments have facilitated the survival of ovarian cancer patients or improved their prognosis, which may increase the overall survival rate of cancer patients. For example, an increase in overall survival was reported with surgical paradigm shifts for more complete surgical cytoreduction [19,20]. Systematic treatment, including platinum-based chemotherapy and targeted therapies, such as angiogenesis inhibitor and Poly ADP-ribose polymerase (PARP) inhibitor, also improved the prognosis of ovarian cancer patients [21]. Thirdly, improving the survival of cancer patients also relies on early detection. The five-year survival for early stage patients is 80%, compared to 25% for advanced stages [36]. In the National Cervical Cancer Screening Program, concurrent vaginal ultrasound can detect ovarian cancer earlier [22]. Serum CA125, combined with transvaginal ultrasound, is the most common strategy for ovarian cancer early detection [37]. However, some scientists disputed that the current screen strategies lead to no mortality reduction and cannot be recommended [38]. The new strategy of detecting inherited predisposition, such as the BRCA gene, was considered effective for susceptibility identification [39]. However, whether BRCA genetic testing can improve survival has not been validated in the population to date. Further research is needed to develop effective early screening regimens for ovarian cancer. Fourth, Taizhou is a coastal city in Zhejiang Province in eastern China with rapid economic development, extensive health insurance system coverage, and a high level of medical coverage for residents. The improved survival rate of ovarian cancer patients may also be related to the high level of local education, with a high awareness of core knowledge of Zhejiang residents about malignancy prevention and treatment [25]. People who are aware of knowledge related to malignant cancer prevention and treatment are likely to be concerned about their own health compared to others, and thus they may be more active in early diagnosis and treatment and primary prevention of cancers.

A significant advantage of our study is that it was the first time we implemented the period analysis approach to provide the latest (2014–2018) five-year RS for ovarian cancer patients in Taizhou, Eastern China. In addition, our analysis of the survival trend revealed that the five-year RS of ovarian cancer patients showed an upward trend from 2004–2018. However, we also have limitations: firstly, population-based cancer registries do not usually include clinical information about the staging (e.g., FIGO), histology, and treatment of cancer patients, which adds to the limitations of this study. Secondly, due to the heterogeneity of ovarian cancer, survival varies considerably based on histological type (the endometrioid vs. the serous) and stage of diagnosis (stage I vs. stage IV) [40,41]. Our analysis obscures these differences by lacking data in this regard. In addition, our sample size is relatively small. We only evaluated the latest survival data of ovarian cancer patients in Taizhou, Eastern China, and we could not conduct a broader and more comprehensive evaluation. Therefore, it is necessary to cooperate with provincial or national units if further investigation is to be carried out.

## 5. Conclusions

In this study, we used the period analysis approach for the first time to evaluate the five-year RS of the latest (2014–2018) ovarian cancer patients. The overall five-year RS tended to increase over time, with differences in RS between age at diagnosis and regions. Our timely data on five-year RS of ovarian cancer patients in Taizhou, eastern China, provide a good data base for ovarian cancer surveillance and prevention, with important implications for timely assessment early detection and screening programs for ovarian cancer. 

## Figures and Tables

**Table 1:** Characteristics of ovarian cancer patients diagnosed during 2004–2018 in Taizhou, eastern China.

Characteristics	Number of Cases (%)	Diagnosed Interval			*χ^2^*	*p*
		2004–2008 (%)	2009–2013 (%)	2014–2018 (%)		
Total	770 (100)	53 (100)	290 (100)	427 (100)		
Region						
Urban area	185 (24.0)	25 (47.2)	78 (26.9)	82 (19.2)		
Rural area	585 (76.0)	28 (52.8)	212 (73.1)	345 (80.8)	22.301	<0.0001
Average age (years)	54.4	57.9	55.8	53.2		
Age at diagnosis (years)						
<55	174 (22.6)	9 (17.0)	68 (23.4)	97 (22.7)		
55~64	164 (21.3)	10 (18.9)	63 (21.7)	91 (21.3)	4.858	0.5621
65~74	196 (25.5)	14 (26.4)	81 (27.9)	101 (23.7)		
>74	236 (30.6)	20 (37.7)	78 (26.9)	138 (32.3)		

**Table 2:** The five-year relative survival of ovarian cancer patients during 2014 to 2018 in Taizhou, eastern China.

	Estimated Value (%)	Standard Error (SE)
Total	69.2	1.8
Age at diagnosis (years)		
<55	79.6	3.6
55~64	72.9	3.0
65~74	68.4	2.8
>74	66.9	2.3
Region		
Urban area	77.5	3.6
Rural area	64.9	2.4

## Data Availability

The raw data supporting the conclusions of this article will be made available upon reasonable request to the corresponding author (T.C.). The data are not publicly available due to privacy or ethical restrictions.
